# *Chlamydia pneumoniae* Infection Induced Allergic Airway Sensitization Is Controlled by Regulatory T-Cells and Plasmacytoid Dendritic Cells

**DOI:** 10.1371/journal.pone.0020784

**Published:** 2011-06-10

**Authors:** Timothy R. Crother, Nicolas W. J. Schröder, Justin Karlin, Shuang Chen, Kenichi Shimada, Anatoly Slepenkin, Randa Alsabeh, Ellena Peterson, Moshe Arditi

**Affiliations:** 1 Pediatrics Infectious Diseases, Cedars-Sinai Medical Center, University of California Los Angeles, Los Angeles, California, United States of America; 2 Department of Pathology, University of California Irvine, Irvine, California, United States of America; 3 Department of Pathology and Laboratory Medicine, Cedars-Sinai Medical Center, University of California Los Angeles, Los Angeles, California, United States of America; Tulane University, United States of America

## Abstract

*Chlamydia pneumoniae* (CP) is associated with induction and exacerbation of asthma. CP infection can induce allergic airway sensitization in mice in a dose- and time-dependent manner. Allergen exposure 5 days after a low dose (mild-moderate), but not a high dose (severe) CP infection induces antigen sensitization in mice. Innate immune signals play a critical role in controlling CP infection induced allergic airway sensitization, however these mechanisms have not been fully elucidated. Wild-type, TLR2−/−, and TLR4−/− mice were infected intranasally (i.n.) with a low dose of CP, followed by i.n. exposure to human serum albumin (HSA) and challenged with HSA 2 weeks later. Airway inflammation, immunoglobulins, eosinophils, and goblet cells were measured. Low dose CP infection induced allergic sensitization in TLR2−/− mice, but not in TLR4−/− mice, due to differential Treg responses in these genotypes. TLR2−/− mice had reduced numbers of Tregs in the lung during CP infection while TLR4−/− mice had increased numbers. High dose CP infection resulted in an increase in Tregs and pDCs in lungs, which prevented antigen sensitization in WT mice. Depletion of Tregs or pDCs resulted in allergic airway sensitization. We conclude that Tregs and pDCs are critical determinants regulating CP infection-induced allergic sensitization. Furthermore, TLR2 and TLR4 signaling during CP infection may play a regulatory role through the modulation of Tregs.

## Introduction

Asthma is characterized by an inappropriate immune response that results in bronchoconstriction, mucus secretion, and eosinophilic airway inflammation, and is thought to develop in two stages [1]. The first stage, known as sensitization, encompasses the exposure to a normally innocuous antigen in the lungs during some type of inflammatory response that leads to the development of T_h_2 type memory cells [2]. Later, upon re-exposure to the same antigen, these memory cells are activated, resulting in an inflammation of the lungs. There are many possible mechanisms for antigen sensitization to occur, and one increasingly important scenario involves respiratory infections.

It is known that respiratory viral infections among young children can lead to a much greater risk of asthma development [3]. Experimental studies using murine models have also shown that pulmonary viral infections can enhance antigen sensitization and or lead to exacerbation of asthma, depending on the timing and severity of infection [4], [5]. However, much less is known about the interactions of bacterial infections and asthma. *Chlamydia muridarum* infection in the lungs of neonatal mice results in a more severe asthma phenotype later on in life [6] and many clinical studies have linked the bacterial pathogen, *Chlamydia pneumoniae* (CP) with both the development and exacerbation of asthma [7], [8], [9]. Murine studies showed that a mild pulmonary CP infection could act as an adjuvant for antigen sensitization to an otherwise inert protein (human serum albumin (HSA)), which upon re-exposure to HSA resulted in eosinophilic airway inflammation and goblet cell hyperplasia [10]. Interestingly, allergic airway sensitization critically depended on the severity and timing of CP infection, as a low-dose (mild) infection and antigen exposure within 5 days of infection induced allergic sensitization, whereas high-dose (severe) CP infection or antigen exposure 10 days after infection did not [10]. Temporal and dose-related effects on the ability of CP infection to induce allergic sensitization reflected DC activation and could be reproduced by means of adoptive transfer of HSA-pulsed lung DCs from infected mice, and be modulated by Treg cells [10].

In this study, we now provide additional mechanistic insights on the specific roles of Treg cells and plasmacytoid DCs in the temporal and dose-related allergic sensitization induced by CP infection. We show that TLR4 signaling is required for antigen sensitization, but TLR2 is not, and that Tregs are involved in both phenotypes. Additionally, we find that during a severe CP infection, which is normally non-permissive for allergic sensitization in this murine model, both Tregs and plasmacytoid dendritic cells (pDCs) are increased in the lung and that the depletion of either cell type results in the reversal of the phenotype and allows development of allergic sensitization. In further mechanistic investigations we observed that in addition to a live CP infection, UV killed CP (CPUV) can also induce allergic sensitization and we show that this observation is not specific to pulmonary CP infection but can occur with other bacteria as well, as UV killed *Bordetella bronchiseptica* was also able to induce allergic sensitization to HSA. Indeed, we show that bacterial ligands, such as a TLR2 ligand, in addition to the TLR4 ligand LPS, are able to induce the allergic sensitization. Collectively these data now provide strong evidence that killed CP, or other bacteria such as *Bordetella bronchiseptica*, as well as TLR2 ligands can induce allergic sensitization, and that during a live CP infection, both Tregs and pDCs critically regulate the temporal and dose-dependent induction of allergic airway sensitization. Additionally, TLR2 and TLR4 signaling during CP infection may play a regulatory role though the modulation of Tregs.

## Results

### UV killed C. pneumoniae and UV killed Bordetella bronchiseptica induce antigen sensitization and eosinophilic airway inflammation

**Figure 1:**
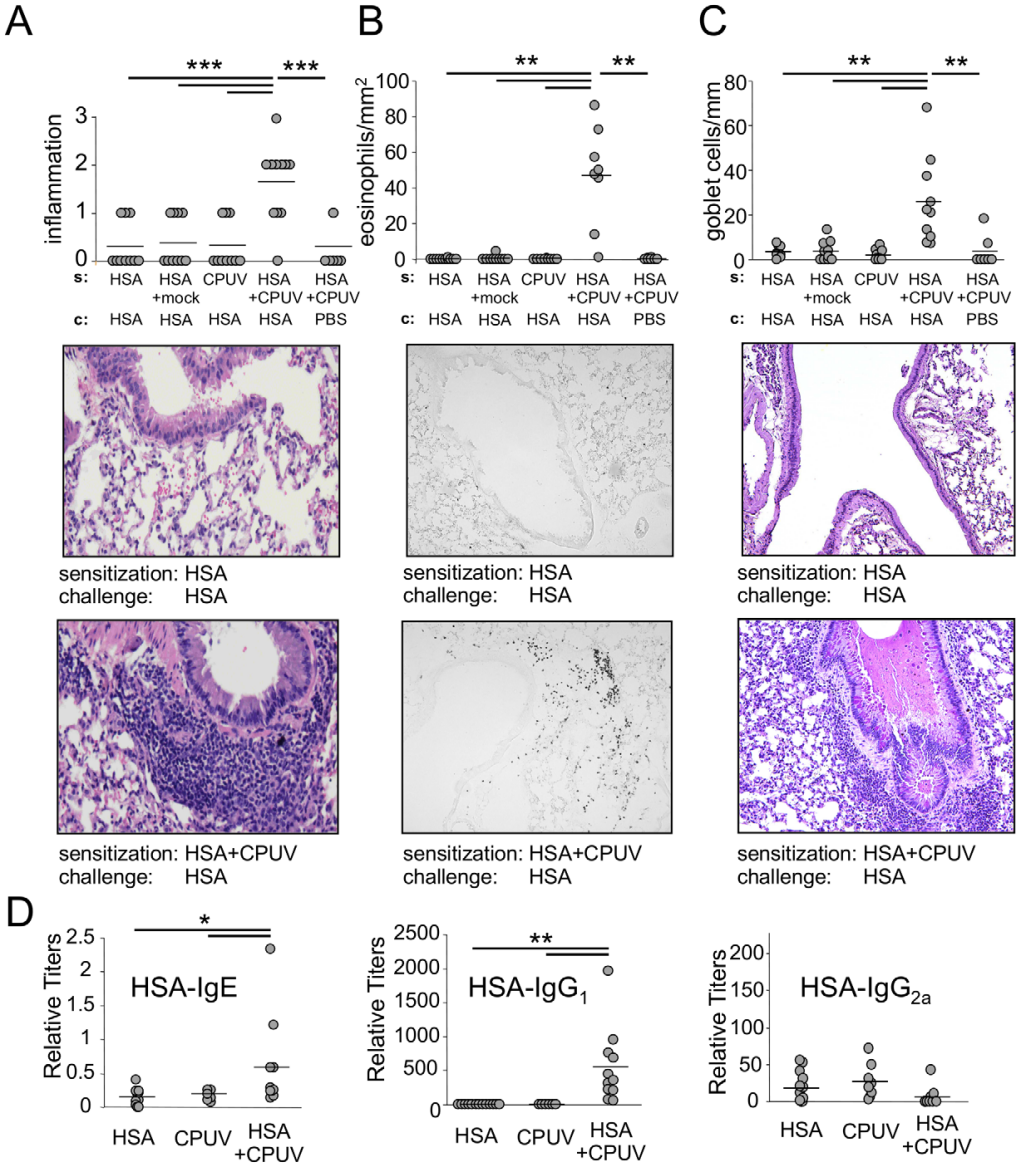
Parallel exposure of mice to CPUV and HSA induces allergic airway sensitization. **A:** Inflammatory scores of H&E stained lung sections of mice after sensitization and challenge. Shown below are representative sections. **B:** Staining of lung sections for eosinophil-specific peroxidase. Total numbers of eosinophils were related to the total area of the section. Shown below are representative sections. **C:** Goblet cells visualized by PAS staining. Total numbers of goblet cells were related to the total length of bronchial basal membrane in the section. Representative sections are shown below. **D:** HSA-specific IgE, IgG1 and IgG2a titers. *p≤0.05, **p≤0.01, ***p≤0.001.

We wished to investigate if in addition to live CP infection [10], UV killed CP (CPUV) could also induce allergic sensitization and if so, try and break down the relative importance of various PAMP signals during infection towards antigen sensitization. Mice were exposed to 1×10^6^ CPUV in combination with 100 µg human serum albumin (HSA) or PBS control intranasally, then re-exposed to HSA two weeks later (Fig S1A). Upon re-exposure to HSA, mice that were initially exposed to HSA and CPUV developed significant inflammation in the lungs as determined by H&E staining (Figure 1A). In contrast, mice that received HSA without CPUV during sensitization, or mice that were challenged with PBS instead of HSA did not develop pulmonary inflammation. Similarly, mice sensitized with HSA and CPUV, followed by HSA challenge, had significantly increased eosinophils in the lung compared to controls (Figure 1B). Significant goblet cell hyperplasia was also found in mice that received both HSA and CPUV during sensitization, and HSA challenge (Figure 1C). Finally, HSA-specific IgE and IgG1 levels were significantly increased in mice that were sensitized with both HSA and CPUV, while IgG2a levels were unchanged (Figure 1D). These data indicate that CPUV can also act as a potent adjuvant and drive antigen sensitization. We next wanted to determine if this observation was specific to CPUV or if it could be induced with another bacterial adjuvant. We used UV killed *Bordetella bronchiseptica* (BBUV). Mice were exposed to 1×10^6^ CFU BBUV in combination with 100 µg human serum albumin (HSA) or PBS control intranasally, then re-exposed to HSA two weeks later as described above. Upon re-exposure to HSA, mice that were initially exposed to HSA and BBUV also developed significant inflammation in the lungs as well as significantly increased infiltration of eosinophils, goblet cells, and HSA-specific IgE and IgG1 (Fig S2A–D), similar to findings obtained by CPUV. These observations clearly suggest that killed bacteria other than CP can also induce allergic sensitization, implying that various microbial ligands present in killed bacteria are most likely able to drive this antigen sensitization.

### TLR2 signaling can drive antigen sensitization

**Figure 2:**
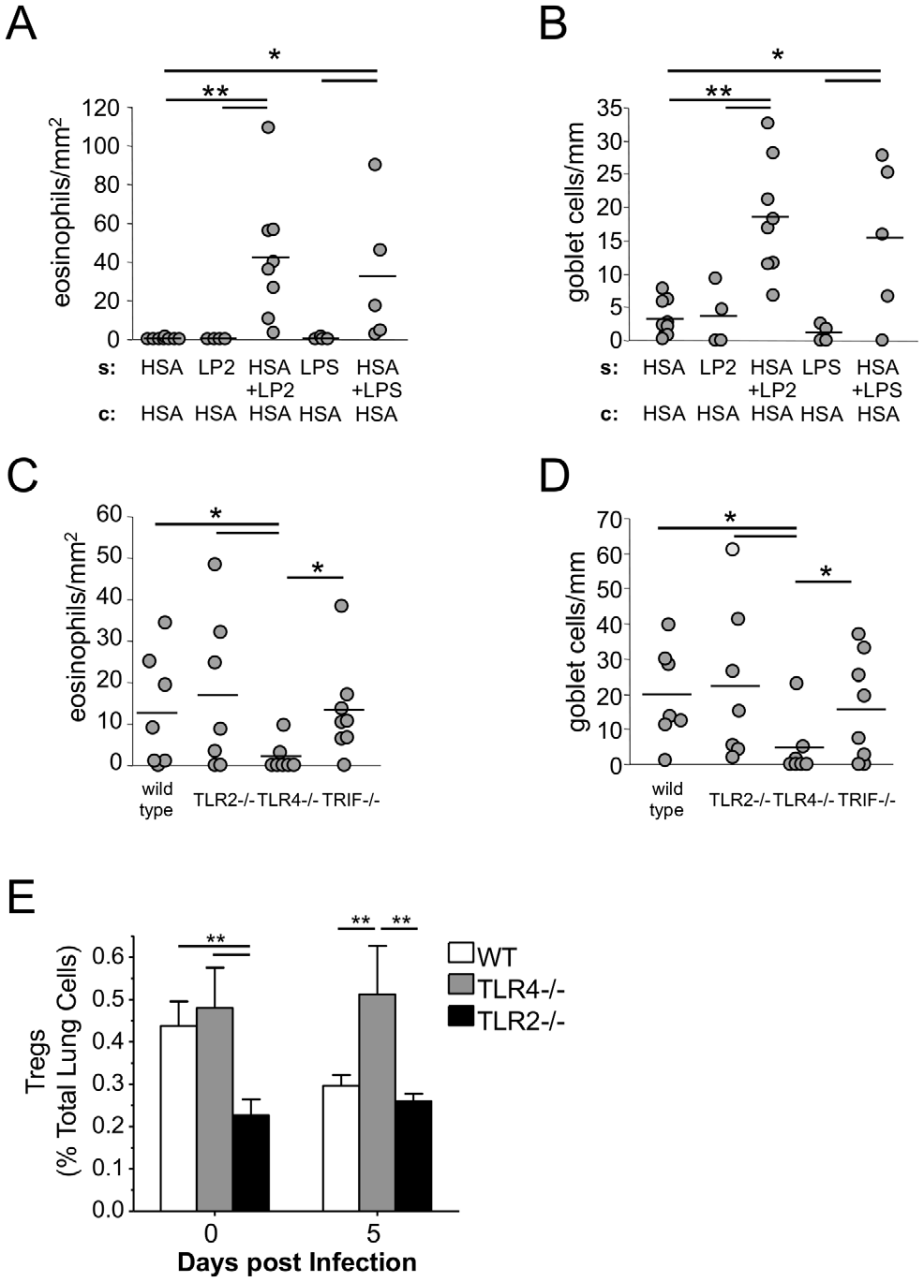
The effects of TLR signaling on CP infection induced antigen sensitization. **A and B:** Administration of HSA to mice in the presence of TLR2- or TLR4-ligands induces airway sensitization. Mice were sensitized with either HSA alone (n = 8), 100 ng Pam2CSK4 (LP2) alone (n = 4), HSA+100 ng LP2 (n = 8), 100 ng LPS alone (n = 4) or HSA+100 ng LPS (n = 5). Mice were challenged with HSA and eosinophil and goblet cell numbers were assessed. **C and D:** Wild type (WT) (n = 7), TLR2−/− (n = 7), TLR4−/− (n = 7) and TRIF−/− (n = 8) mice were infected with 5×10^5^ CP followed by HSA sensitization and challenge as shown in Figure 1B. Eosinophil and goblet cell numbers were assessed. **E:** Percentage of lung Tregs in wild type, TLR2−/−, and TLR4−/− uninfected and CP-infected mice. Wild type, TLR4−/−, and TLR2−/− mice (n = 3–6 per group) were inoculated with 5×10^5^ IFU of CP. 5 days after inoculation, mice were sacrificed, lung leukocyte preparations were generated and analyzed for the presence of CD4+, CD25+, Foxp3+ Tregs by flow cytometry. Data are presented as a percentage of total lung cells. Shown are combined data from 2–3 separate experiments. *p≤0.05, **p≤0.01.

It is known that innate immune cells can detect *Chlamydia pneumoniae* by both TLR2 and TLR4 pattern recognition receptors (PRR) [11], [12]. However, *C. pneumoniae* uses predominantly TLR2 signaling for cytokine release [12], [13]. MyD88 signaling is essential for CP infection induced antigen sensitization in this allergic asthma model [10]. Both TLR2 and TLR4 signaling can proceed through MyD88, and since the role of a TLR4 ligand, LPS alone in this antigen sensitization model has already been described [14], we investigated whether TLR2 signaling by itself could also induce antigen sensitization in this model. Using the same protocol as above, except substituting CPUV with either LP2 (TLR2 ligand) or LPS (TLR4 ligand), upon subsequent challenge with HSA, mice that were sensitized in the presence of either LPS or LP2 developed both eosinophilic airway inflammation and goblet cell hyperplasia (Figure 2A–B), while the control mice did not. As far as we are aware, this is the first reported instance of a TLR2 ligand driving allergic antigen sensitization in the lung.

### Airway sensitization by C. pneumoniae requires TLR4- but not TLR2- dependent signaling

We next investigated the roles of TLR2 and TLR4 signaling during live CP infection induced antigen sensitization. Since CP signals predominantly through TLR2 for cytokine release, and that a TLR2 ligand, LP2 alone could drive antigen sensitization, we hypothesized that CP infection-induced allergic sensitization would not be seen in TLR2−/− mice, but would be observed in TLR4−/− mice. Utilizing our previously published protocol for live infection induced antigen sensitization (Fig S1B) [10], WT, TLR2−/−, TLR4−/−, and TRIF−/− mice were infected with 5×10^5^ IFU of CP, sensitized with HSA for three days (beginning 5 days after infection), and then challenged with HSA. As expected WT mice developed allergic sensitization with eosinophilia and goblet cell hyperplasia in the lungs of mice that were infected with CP and sensitized/challenged with HSA (Figure 2C–D). However, we observed that TLR2−/− mice were successfully sensitized, while TLR4−/− mice were not. Indeed, TLR2−/− mice developed increased eosinophils and goblet cells in the lung, while TLR4−/− mice did not (Figure 2C–D), suggesting that CP-induced allergic sensitization requires TLR4 but not TLR2. TLR4 can signal through both MyD88 and TRIF. MyD88 signaling is absolutely required for CP infection induced antigen sensitization [10], but the role of TRIF signaling is unknown. Therefore we investigated whether TRIF−/− mice could be sensitized by live CP infection. We observed that TRIF was not important in infection-induced allergic sensitization as TRIF−/− mice developed allergic sensitization and were indistinguishable from WT mice (Figure 2C–D). In order to dissect the molecular mechanisms as to why TLR2−/− mice but not TLR4−/− mice could be sensitized, we investigated the relative activation of TLR2−/− and TLR4−/− bone marrow derived dendritic cells (BMDCs) by CP infection. BMDCs were exposed to live CP, LPS, LP2, or CPUV for 24 hrs and IL-6 levels in the supernatants were measured. LPS, LP2, CP, and CPUV all induced IL-6 production in WT BMDCs, as expected (Fig S3A). In BMDCs obtained from TLR4−/− mice, LPS did not induce IL-6, LP2 exposure was unchanged from wild type mice, while both live and UV killed *C. pneumoniae* still induced IL-6, but at lower levels than WT cells. On the other hand, TLR2−/− BMDCs responded to LPS, and but not to the TLR2 ligand LP2 as expected, but showed substantially reduced IL-6 release in response to live-CP and to CPUV compared to WT and TLR4−/− BMDCs. While these data corroborate previously published data that CP signals mainly through TLR2 for cytokine release, they do not explain the lack of sensitization in TLR4−/− mice. We next investigated the activation state of the BMDCs after exposure to CP in order to discern any differential responses between TLR2- and TLR4-deficient BMDCs. Both TLR2−/− and TLR4−/− BMDCs had reduced levels of CD80 and CD86 expression on the surface compared to WT BMDCs after CP exposure (Fig S3B). Again, these data did not provide any explanation for the differential sensitization observed between TLR2−/− and TLR4−/− mice. We next studied the role of Tregs in these two genotypes. Previous reports revealed that TLR2−/− mice had reduced circulating numbers of Tregs [15], [16]. Since Tregs play an important suppressive role in the development of asthma [17], [18] we therefore investigated the numbers of Tregs in these mice during infection. WT, TLR2−/− and TLR4−/− mice were infected with CP as before and the numbers of Tregs in the lung were assessed by flow cytometry 5 days after infection. We observed that baseline levels of Tregs in the lung were significantly reduced in TLR2−/− mice compared to WT and TLR4−/− mice (Figure 2E, Figure S4A), which was maintained at 5 days after infection, during the time when sensitization would begin. Additionally, TLR4−/− mice now had a significant increase in Tregs as compared to WT mice (Figure 2E). The reduced numbers of Tregs in TLR2−/− mice provide a possible explanation for the ability of TLR2−/− mice to be sensitized, despite reduced activation and responsiveness in their dendritic cells. Conversely, the inability of TLR4−/− mice to be sensitized during CP infection may be a result of increased levels of Tregs in their lungs.

### Treg depletion in TLR4−/− mice allows allergic sensitization by C. pneumoniae infection

**Figure 3:**
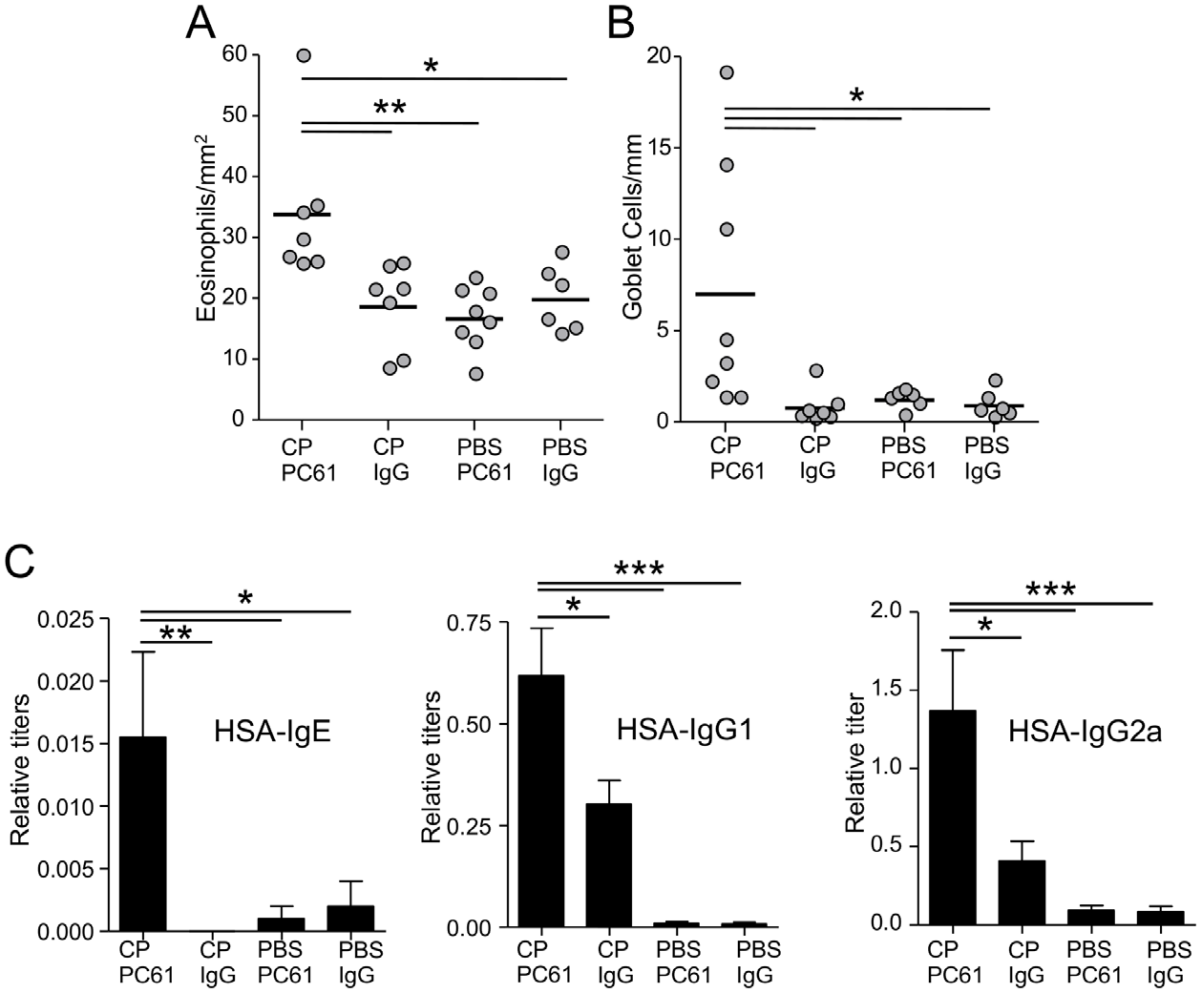
Treg depletion allows CP infection induced antigen sensitization in TLR4−/− mice. TLR4−/− mice received either CP+PC61 mAB (n = 8), CP+IgG control (n = 7), PBS+PC61 mAB (n = 8), or PBS+IgG control (n = 6), and were sensitized and challenged with HSA as described in Figure 1C. **A:** Staining of lung sections for eosinophil-specific peroxidase. Total numbers of eosinophils were related to the total area of the section. **B:** Goblet cells visualized by PAS staining. Total numbers of goblet cells were related to the total length of bronchial basal membrane in the section. **C:** HSA-specific IgE, IgG1, and IgG2a titers. *p≤0.05, **p≤0.01, ***p≤0.001.

Since the infected TLR4−/− mice had increased levels of Tregs in the lungs during the sensitization period (Figure 2E), we decided to directly test whether these Tregs were preventing allergic sensitization. Using the same model as above, TLR4−/− mice were given the PC61 antibody (or IgG control) one day prior to sensitization to deplete the Tregs during the sensitization window. In agreement with our hypothesis, the depletion of Tregs in TLR4−/− mice reversed the phenotype and allowed allergic sensitization with eosinophilia and goblet cell hyperplasia in these mice (Figure 3A–B). Additionally, HSA specific IgE and IgG1 were also significantly increased in these mice (Figure 3C). Importantly, in a control experiment, uninfected mice that received the PC61 antibody during sensitization did not develop an allergic airway response to HSA. These observations suggest that Tregs prevent antigen sensitization in TLR4−/− mice infected with CP.

### TLR2−/− mice have an extended time-window for C. pneumoniae infection induced antigen sensitization

**Figure 4:**
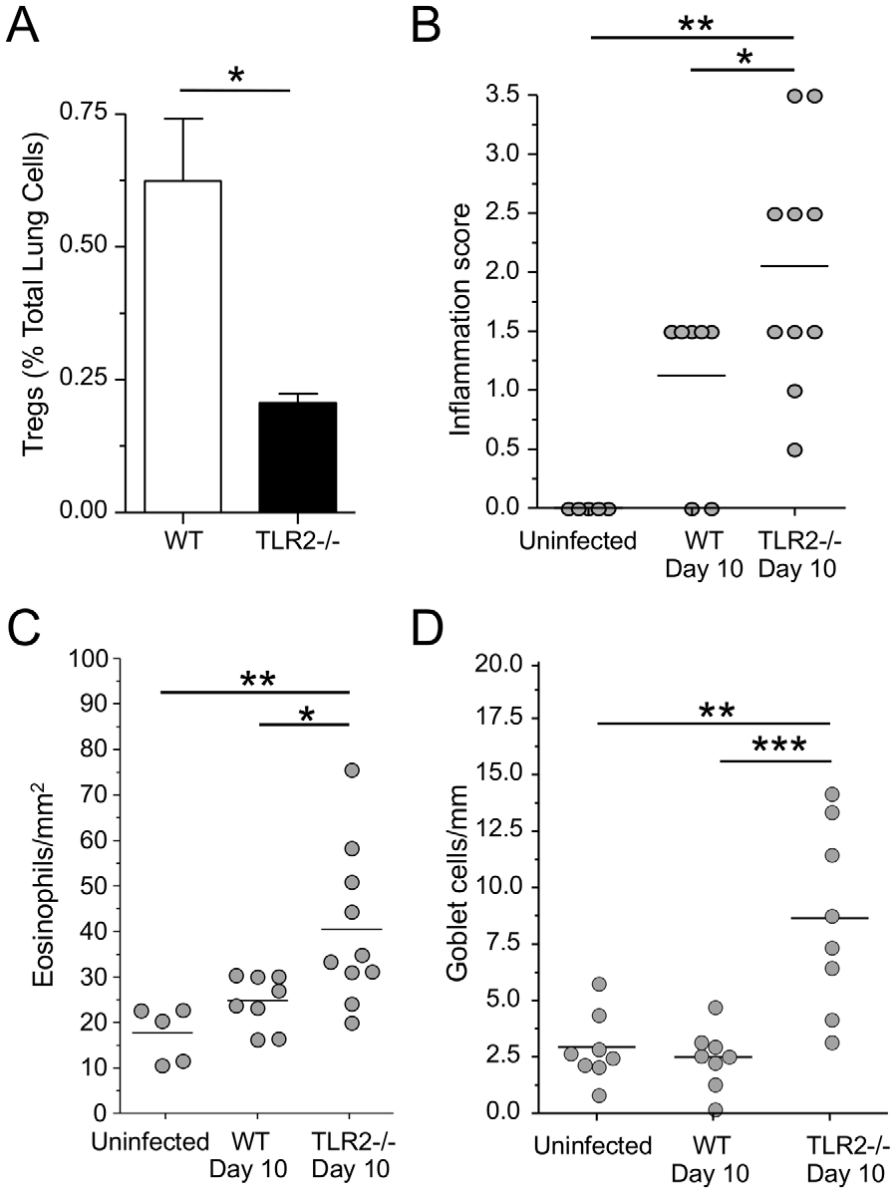
TLR2−/− mice have an extended time window for CP infection induced antigen sensitization. **A:** Tregs were assessed in the lungs of WT and TLR2−/− mice 10 days after low dose (5×10^5^) CP infection. Data are presented as CD4+, CD25+, FoxP3+ Tregs as percentage of total lung cells. **B–D:** WT infected mice (n = 8), and TLR2−/− mice infected (n = 8) and uninfected (n = 8) were sensitized and challenged with HSA as described in Figure 1D. Inflammation (**B**), Eosinophils (**C**) and Goblet cells (**D**) were scored for as before. *p≤0.05, **p≤0.01, ***p≤0.001.

Tregs control the time window during which WT mice could be sensitized relative to the start of the CP infection [10]. Normally, WT mice could be sensitized 5 days after low-dose (mild) infection, but not 10 days after, due to an influx of Tregs [10]. However, if the Tregs were depleted, the mice could now be sensitized 10 days after infection [10]. We reasoned that since TLR2−/− mice had reduced numbers of lung Tregs at baseline, and did not increase their Treg numbers 5 days after infection, they should be able to develop sensitization even at day 10 after infection if Treg played a critical role in this event. We infected TLR2−/− and WT mice as before and sensitized them with HSA 10 days after infection instead of 5 days (Fig S1B). We observed that TLR2−/− mice had significantly reduced Tregs 10 days after infection compared to WT mice (Figure 4A, Figure S4B). As we predicted, we observed that TLR2−/− mice can develop allergic sensitization when the antigen is introduced initially both at day 5 and day 10 after infection, while WT mice is sensitized only when antigen is given at day 5 but not day 10 after infection (Figures 4B–D and 2C–D). Importantly, in control experiments, uninfected TLR2−/− mice were not sensitized, indicating that while the Treg numbers are also reduced in these mice, CP infection was still required as an adjuvant for antigen sensitization to occur. These observations, while not providing definitive proof, strongly suggest that the reduced numbers of Tregs in TLR2−/− mice likely allowed for the extended time window for allergic sensitization and support the conclusion that Tregs critically regulate infection-induced allergic sensitization.

### Severe C. pneumoniae infection increases Tregs and pDCs in the lung

**Figure 5:**
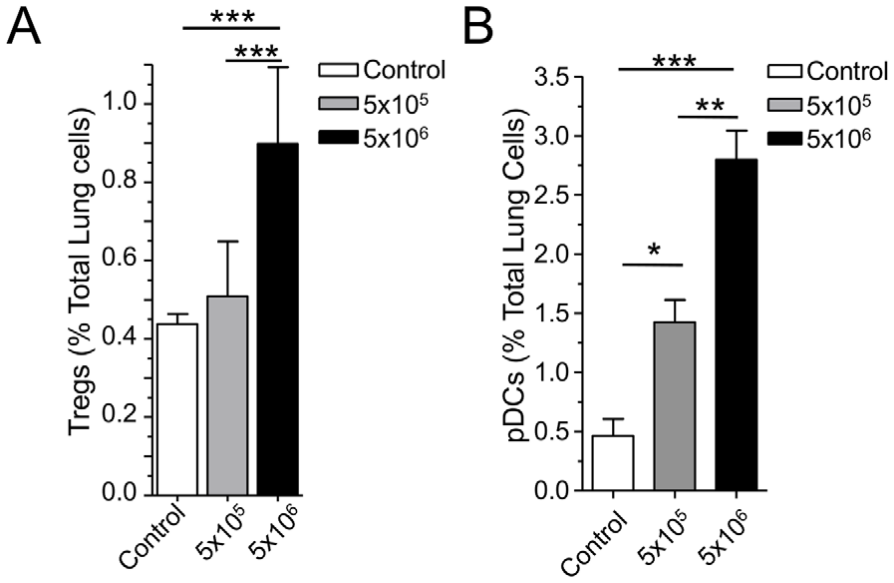
Tregs and pDCs are induced during high dose CP infection. **A–C:** WT mice were infected with either low dose (5×10^5^) or high dose (5×10^6^) CP intranasally. 5 days after infection, the lungs were harvested and single cell suspensions were analyzed by flow cytometry. **A:** Tregs. Data are presented as CD4+, CD25+, FoxP3+ Tregs as a percentage of total lung cells. **B:** pDCs. Data are presented as BST2+, B220+, CD3 CD19 CD11b- and side scatter low as a percentage of total lung cells. *p≤0.05, **p≤0.01, ***p≤0.001.

In our earlier publication, we reported that dose-dependence or infection severity was a determinant for antigen sensitization [10]. A moderate low dose infection (5×10^5^ IFU) induced antigen sensitization, while a severe high dose infection (5×10^6^ IFU) prevented sensitization from occurring. Given the importance of Tregs during CP infection-induced antigen sensitization, we investigated whether Tregs might also play an inhibitory role during a severe (high-dose) CP infection. We infected WT mice with either 5×10^5^ or 5×10^6^ IFU CP and examined the number of Tregs in the lungs 5 days after infection by flow cytometry. There was no significant change in Treg numbers in lungs between uninfected mice and low dose infected mice at day 5 (Figure 5A). However, we observed a dramatic and significant increase of Tregs in the lungs of mice in the high-dose infection group, compared to low-dose infection group and uninfected control mice (Figures 5A, Figure S4C). We also investigated Plasmacytoid Dendritic cells (pDCs), another cell type that plays a suppressive role towards antigen sensitization. Like Tregs, pDCs were significantly increased during a severe CP infection compared to uninfected mice (Figure 5B, Figure S4D). However, the pDCs were also increased in the low dose infection group as well, although not as much as with the high dose infection. For representative flow cytometric scatterplots of Tregs and pDCs during infection, see Figure S4E–F. Thus two suppressive cell types towards antigen sensitization, Tregs and pDCs, were present in greater numbers during a severe CP infection in WT mice, compared to the moderate infection group, likely preventing antigen sensitization.

### Treg depletion allows antigen sensitization to occur during severe C. pneumoniae infection

**Figure 6:**
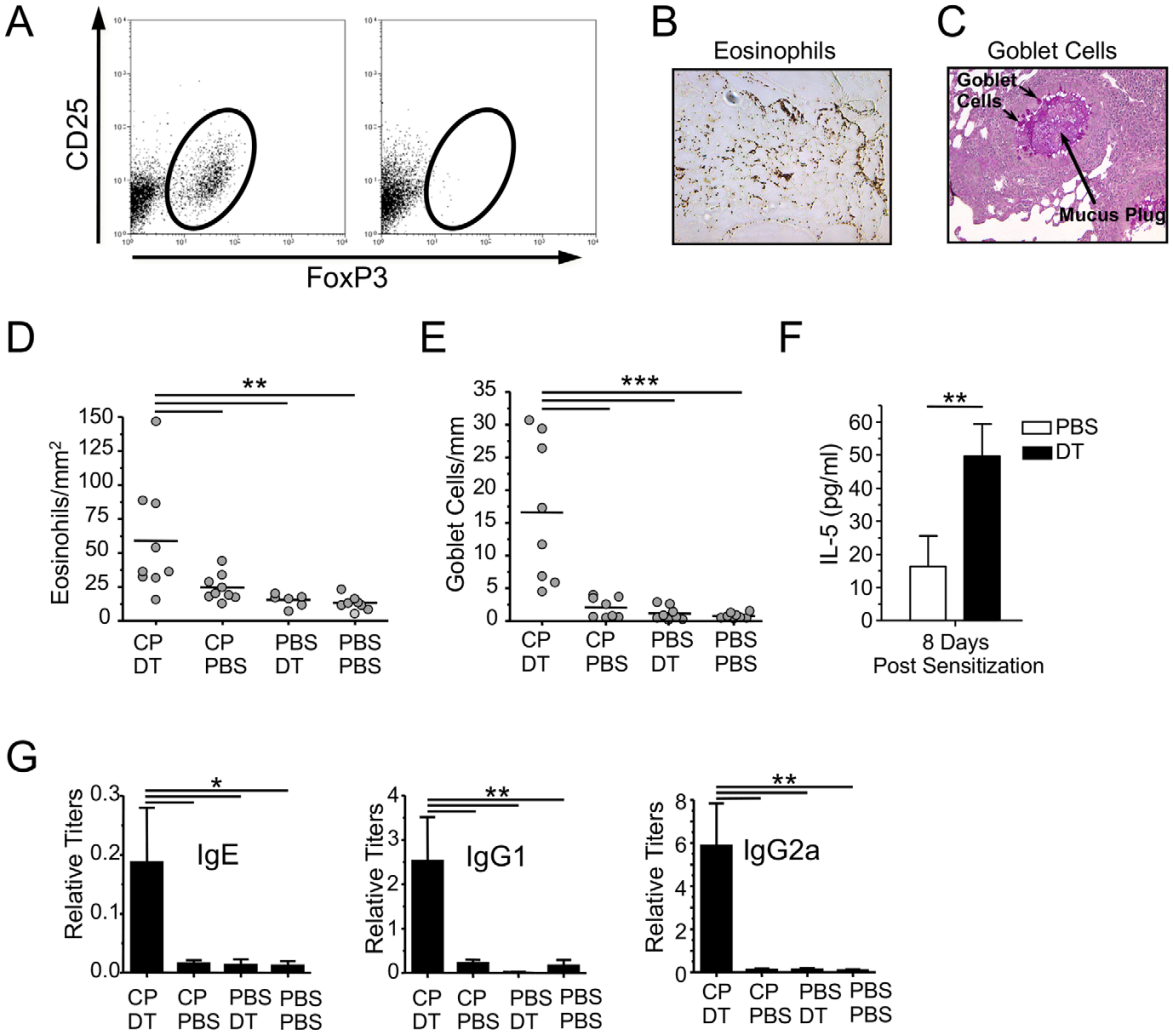
Treg depletion allows high dose CP infection induced antigen sensitization. FoxP3-DTR tg mice received either CP+diphtheria toxin (DT) (n = 9), CP+PBS (n = 9), PBS+DT (n = 7), or PBS+PBS (n = 7), and were sensitized and challenged with HSA as described in Figure 1E. **A:** Representative flow cytometric analysis of Treg depletion the day after DT injection. **B:** Representative staining of lung eosinophils in CP infected and DT treated mice. **C:** Representative staining of lung goblet cells in CP infected and DT treated mice. **D:** Total numbers of eosinophils were related to the total area of the section. **E:** Total numbers of goblet cells were related to the total length of bronchial basal membrane in the section. **F:** Mediastinal lymph node cells were restimulated with HSA after sensitization. Supernatants were measured for IL-5 by ELISA. **G:** HSA-specific IgE, IgG1, and IgG2a titers. *p≤0.05, **p≤0.01, ***p≤0.001.

In order to determine if the increased Tregs in the lung during a severe CP infection played a critical role in preventing antigen sensitization, we depleted Tregs during the course of sensitization. Specific depletion of Tregs was made possible by the use of the Foxp3-specific human diphtheria toxin receptor (DTR) transgenic mice (FoxP3-DTR-tg), in which all Foxp3 Treg cells express human diphtheria toxin (DT) receptor (DTR) and, therefore can be efficiently eliminated upon treatment with DT [19], [20]. FoxP3-DTR-tg mice were infected with high dose (5×10^6^ IFU) CP and sensitized 5 days later as before. However, at one day before and on the second day of sensitization, 1 µg of diphtheria toxin (DT), or PBS control, was injected i.p. into the mice to selectively deplete Tregs (Fig S1C) during sensitization. The mice were then challenged and sacrificed as before. Lung Treg depletion was nearly 100% one day after DT injection as shown in Figure 6A by flow cytometry. Specific depletion of Treg cells during the sensitization period in high-dose CP- infected FoxP3-DTR-tg mice reversed the phenotype and allowed the high dose infection to result in allergic sensitization with significantly increased recruitment of eosinophils in the lungs (Figure 6B, 6D) as well as goblet cell hyperplasia, compared to infected FoxP3-DTR-tg mice that did not receive DT (Figure 6C, 6E). Both PBS control and uninfected DT controls did not respond to antigen challenge. Thus both infection and Treg depletion was required for sensitization to occur. We isolated mediastinal lymph nodes 8 days after the conclusion of sensitization from infected and Treg-depleted mice and restimulated the lymphocytes with HSA and observed significantly increased IL-5 production in response to antigen stimulation compared to control mice with undepleted Tregs (Figure 6F). Additionally, HSA specific IgE and IgG1 were significantly increased in the serum of the infected and Treg-depleted mice (Figure 6G). Not surprisingly since there was a severe intracellular infection, HSA specific IgG2a, a Th1 isotype, was also increased in these mice (Figure 6G). Thus Treg depletion allowed for the generation of antigen specific Th2 cells that resulted in allergic sensitization and eosinophilic airway inflammation upon restimulation with the same antigen.

### pDC depletion also allows antigen sensitization to occur during severe C. pneumoniae infection

**Figure 7:**
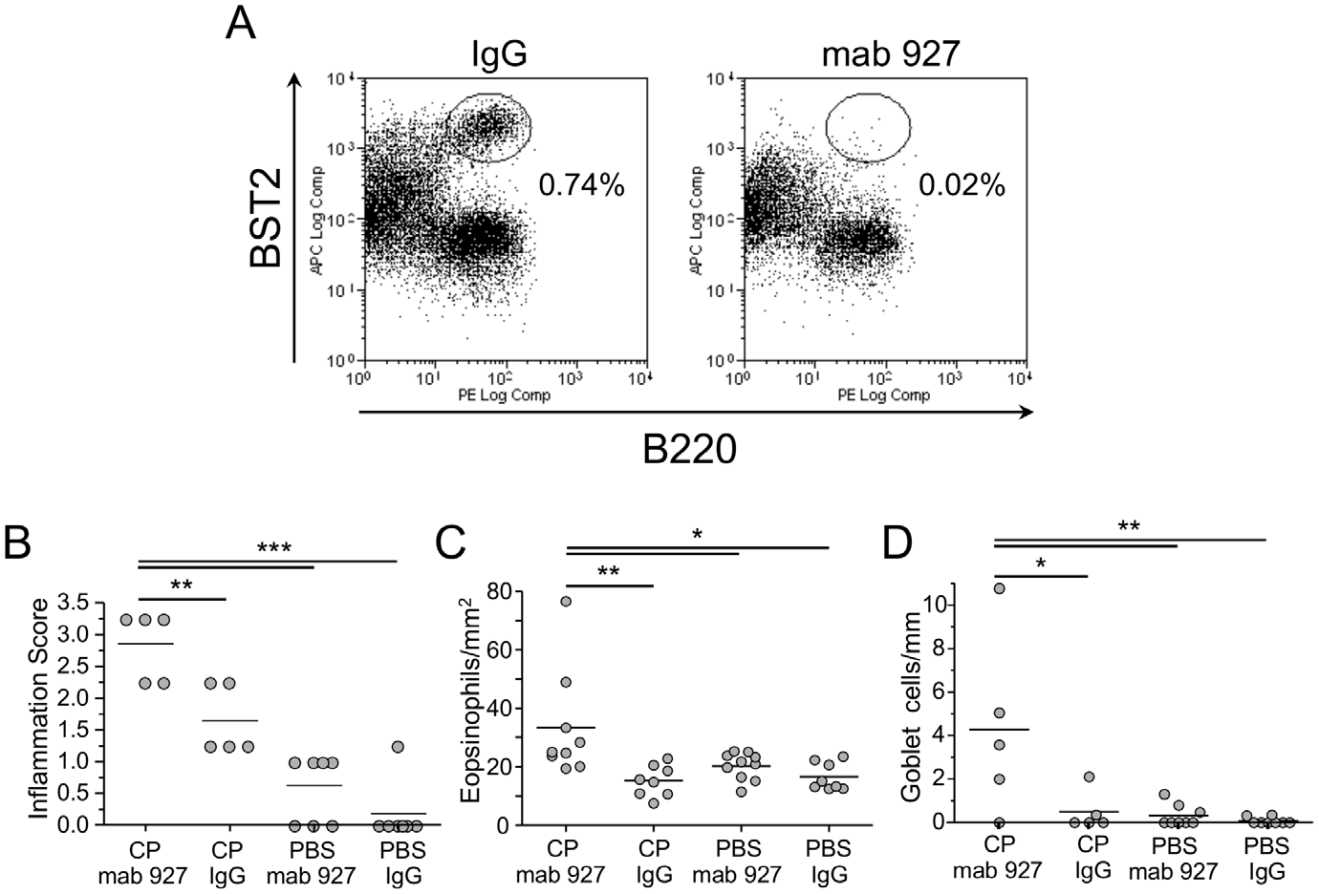
pDC depletion allows high dose CP infection induced antigen sensitization. **A–D:** WT mice received either CP+mAB 927 (n = 9), CP+IgG control (n = 8), PBS+mAB 927 (n = 10), or PBS+IgG control (n = 8), and were sensitized and challenged with HSA as described in Figure 1E. **A:** Representative flow cytometric analysis of pDC depletion the day after mAB 927 injection. pDCs are indicated in the gated population. Inflammation (**B**), Eosinophils (**C**) and Goblet cells (**D**) were scored for as before. *p≤0.05, **p≤0.01, ***p≤0.001.

During high dose CP infection, a condition that does not allow allergic sensitization, we also observed significantly increased pDC numbers in the lungs, in addition to increased number of Tregs, compared to low-dose infection group as shown above (Figure 5B). Given the known interaction between pDCs and Tregs [21], and the fact that pDCs can also have a suppressive effect on allergic sensitization [22], [23], we next sought to determine if the increased pDCs numbers in the lungs observed during high dose CP infection may have also contributed to suppression of antigen sensitization in this group of mice. To test this possibility, we infected WT mice with a high dose of CP and depleted their pDCs using a pDC-specific depleting antibody (mAb927 [24]) (Fig S1C and Figure 7A) during the sensitization window. pDC-depletion allowed the high-dose CP-infected mice to become sensitized compared to non-depleted controls (Figure 7B–D). pDC-depleted uninfected controls did not develop an allergic airway response. Taken together, our data suggest that in addition to Tregs, pDCs can also provide a suppressive signal preventing bacterial pulmonary infection-induced allergic asthma sensitization in case of high-dose infection, and that these two regulatory cell types work in concert to regulate the temporal and dose dependent adjuvant effects of the infection.

## Discussion

*Chlamydia pneumoniae* lung infection can trigger allergic airway sensitization against an inert inhaled antigen, and that this is dependent on the severity and timing of infection [10]. In this study, we observed that in addition to live CP infection, UV killed CP is also able to act as adjuvant and can trigger successful antigen sensitization. UV killed CP provides ligands for both TLR2 and TLR4 [11], [12], [13], and potentially NOD1 and NOD2 [25], which can likely induce sensitization. Indeed, our results using LPS (TLR4 ligand) or LP2 (TLR2 ligand) are in agreement with previous studies showing that either LPS alone or Pam3Cys could provide an adjuvant effect towards antigen sensitization [14], [26]. However, our observation is novel as it shows that a TLR2 ligand could stimulate antigen sensitization in the lung directly, since a previous study used a subcutaneous route for sensitization [26]. We investigated if the observations we made in CP and killed CP could be generalized to other bacterial pathogens and we obtained similar allergic sensitization by UV killed *Bordetella bronchiseptica*, another pathogen that has been associated with wheezing and development of allergic asthma in children. These observations suggest that killed bacteria other than CP can also induce allergic sensitization, suggesting that microbial ligands in killed bacteria are most likely able to trigger this antigen sensitization and play an adjuvant role.

In the current study, we also sought to dissect the role of the TLR2 and TLR4 in *Chlamydia pneumoniae* infection-induced allergic sensitization. We found that both TLR2 and TLR4 can provide the necessary signals in the lung resulting in antigen sensitization. However, during live CP infection, we observed that only TLR2−/− mice, but not TLR4−/− mice could be sensitized. Further investigation revealed a differential Treg response between these two genotypes; TLR2−/− mice showed reduced numbers of Tregs in the lung after CP infection, while TLR4−/− had increased numbers of Tregs. These differential Treg responses likely explain the differential ability for allergic sensitization that we observed between TLR2- and TLR4-deficient mice. Indeed, depletion of Tregs in TLR4−/− mice reversed the phenotype and allowed antigen sensitization in these mice. Furthermore, reduced Treg numbers in TLR2−/− mice, allowed these mice to be sensitized even at day 10 after infection, extending the time window for antigen sensitization relative to the initial infection. These observations show that allergen sensitization during bacterial lung infection is regulated by CD4^+^ CD25^+^ Tregs.

It was previously reported that TLR2−/− mice have reduced numbers of Tregs in the blood [15]. In fact, additional studies have shown that TLR2 is critical for expansion, survival, and proper function of Tregs [16], [27], [28]. These studies provided us with a possible explanation for the differences we saw in sensitization between TLR2−/− and TLR4−/− mice. Indeed, we found similarly reduced numbers of Tregs in the lungs of TLR2−/− mice. Strikingly, we also found that at the time of sensitization (5 days after infection), TLR4−/− mice actually had much greater numbers of Tregs than TLR2−/− or WT mice. To our knowledge, this is the first report of increased Treg numbers in TLR4−/− mice during a pulmonary infection. Some recent studies indicate that TLR4 and TLR2 might have differential effects on Treg numbers. Zhang et al showed that TLR4−/− mice had increased islet allograft survival due to the presence of Tregs [29]. While another group found that TLR4 signaling impaired the expansion of Tregs during a fungal infection [30]. Finally, TLR2−/− and TLR4−/− mice were found to have opposite phenotypes in a spontaneous model of murine arthritis [31]. In that study, TLR2−/− mice had reduced FoxP3 expression, greater IL-17 expression, and increase arthritis severity, while TLR4−/− mice had a lesser severity of arthritis, and lowered IL-17 expression. Taken together, it is clear that TLR signaling can have a profound effect on the Treg compartment of the immune system.

The observation that a severe (high dose) CP infection prevents antigen sensitization was partially attributed to poor DC trafficking to the regional lymph nodes and a greater Th1 response with high dose infection [10]. However, in this study, we also found the numbers of two suppressive cell types, Tregs and plasmacytoid dendritic cells, were present in the lung in much greater numbers during the severe infection. It is not surprising that the numbers of Tregs would increase during the more severe infection, as one of the purposes of Tregs is to dampen the effects of an over-exuberant immune response [32]. While pDCs are known to play an important role in anti-viral responses, they have also been linked to the activation of Tregs [21], [33]. Indeed, depletion of either cell type during severe pulmonary CP infection resulted in restoration of the antigen sensitization phenotype despite the increased Th1 response. Interestingly, the depletion of pDCs during the severe infection did not alter the numbers of Tregs in the lung or draining lymph node (data not shown), indicating that the pDCs suppressive effects were either mediated by influencing the activity of the Tregs, or by some other mechanism. These observations are consistent with prior studies, where the depletion of either Tregs or pDCs in various non-infectious asthma models has resulted in an increased asthma response [22], [23], [34], [35].

While most studies of pulmonary bacterial infections and asthma are directed towards exacerbation, our investigations have been directed towards the development phase of the disease. Several other groups have investigated the role of bacterial infections on antigen sensitization, looking at *Chlamydia muridarum*, *Streptococcus pneumoniae*, or *Porphyromonas gingivalis* as their infection models [36], [37], [38]. However, all these models employ antigen sensitization via the intraperitoneal route using an adjuvant such as alum, and then examine the effects of bacterial infection on that sensitization. We have chosen to examine the infection itself as the potential adjuvant and have found, depending on the severity and timing of the infection, that the same infection can induce or suppress antigen sensitization. Prevention of *C. pneumoniae* infection by vaccination, especially at early ages, might prevent unwanted allergic sensitizations and the development of asthma. Thus the development of a *C. pneumoniae* vaccine could be a desirable endeavor (with other potentially beneficial effects, such as towards COPD and atherosclerosis). At early ages children acquire both bacterial and viral infections and it is of critical importance to understand the potential mechanisms of antigen sensitization that might result in the development of asthma. It is logical that Tregs play such an important role during sensitization and while a pulmonary bacterial infection can provide an adjuvant effect, that same infection can result in reduced or greater numbers of Tregs, which may ultimately control whether allergic sensitization occurs or not.

## Materials and Methods

### Ethics Statement

All animal experiments were performed according to the guidelines and approved protocol (IACUC Protocol #2097) of the Cedars-Sinai Medical Center Institutional Animal Care and Use Committee. Cedars-Sinai Medical Center is fully accredited by the Association for Assessment and Accreditation of Laboratory Animal Care (AAALAC International) and abides by all applicable laws governing the use of laboratory animals. Laboratory animals are maintained in accordance with the applicable portions of the Animal Welfare Act and the guidelines prescribed in the DHHS publication, Guide for the Care and Use of Laboratory Animals.

### Mice

C57BL/6 mice 8 to 12 weeks of age were used throughout the study and were housed under specific pathogen free conditions [10]. In some studies, C57BL/6 mice were acquired from Jackson Labs (Bar Harbor, ME), while in others, WT littermate controls were used. TLR2−/− and TLR4−/− mice (provided by Shizuo Akira, Osaka, Japan) were backcrossed to C57BL/6 background for at least 8 generations and bred at our facility. FoxP3-DTR-tg mice were acquired from Alexander Rudensky (Sloan-Kettering Cancer Center, NY) and were backcrossed to C57BL/6 background for at least 8 generations.

### Infection with C. pneumoniae

*Chlamydia pneumoniae* CM-1 (ATCC, Manassas, VA) was propagated in HEp-2 cells as previously described [11]. HEp-2 cells and *C. pneumoniae* stocks were determined to be free of Mycoplasma contamination by PCR. Mice were intranasally infected with *C. pneumoniae* by inoculating with 40 µl of PBS containing either 5×10^5^ or 5×10^6^ IFU of the microorganism.

### Allergen sensitization and assessment of eosinophilic airway inflammation

Human serum albumin (HSA) (low endotoxin, Sigma, St Louis, Mo) was used as an antigen throughout the study as described earlier [10] in order to reduce the possibility of LPS contamination [39]. HSA on its own does not induce allergic airway sensitization and is completely inert [10], [39]. Mice previously infected with *C. pneumoniae* received 100 µg of HSA in PBS (sensitization) intranasally on 3 consecutive days, starting at various time points after infection, as indicated in the text and figures (Fig S1). Control groups received PBS only. On days 15, 16, 19, and 20 after initial sensitization, mice were re-exposed to 25 µg HSA (challenge) intranasally as above. Mice were sacrificed on day 21 and sera and lungs were harvested. The right lobes of the lungs were fixed in 10% formalin, and paraffin-embedded and hematoxylin and eosin–stained sections were evaluated. The degree of inflammation was scored by a blinded pathologist, as described previously [11]. Goblet cells were detected by means of periodic acid–Schiff staining. For assessment of eosinophilic airway inflammation, the left lobe was fixed in PBS/2% paraformaldehyde/0.2% picric acid, and 7 µm cryosections were prepared. Eosinophils were detected by means of eosinophil peroxidase– specific staining, as described previously [40]. Data were expressed as the number of eosinophils per square millimeter lung section, as well as the number of goblet cells per millimeter of bronchial basal membrane using Image Pro Plus 5.1 Software (Media Cybernetics, Bethesda, MD) to measure the lung area and the bronchial basal membrane length respectively as reported previously [10]. For some experiments, mice were sensitized with HSA plus LPS (100 ng: a protein-free preparation derived from *Escherichia coli* K235, kindly provided by S.N. Vogel, University of Maryland, Baltimore, MD), Pam_2_Cys lipopeptide (100 ng: EMC microcollections, Tübingen, Germany). In some experiments, 1 µg diphtheria toxin (Sigma, MO) was injected i.p. twice, one day before sensitization and on the second day of sensitization, to deplete Tregs in FoxP3-DTR-tg mice. In other experiments using TLR4-deficient mice, 100 µg PC61 antibody (BioLegend, San Diego, CA) was injected i.p. into mice one day before sensitization to deplete Tregs. One injection could deplete Tregs for at least 4 days [10]. For other experiments, 500 µg mAB 927 (pDC depleting mAB 927 was provided to us by Marco Colonna, Washington University, MO) [24] was injected i.p. twice, one day before sensitization and on the second day of sensitization, to deplete pDCs in mice. Rat IgG from serum was used as antibody controls (Sigma, MO).

### Flow cytometry

Total lung cell preparations from mice infected with *C. pneumoniae* were prepared by digesting lungs with 100 µg/mL Blendzyme 3 (Roche, Mannheim, Germany) and 20 µg/mL DNAseI (Roche). Cell suspensions were prepared as published earlier [10]. Numbers of pDCs in the lungs were identified as CD11b CD3 CD19 negative, and B220 BST2 positive. Treg cells in the lungs were identified as CD4 CD25 FoxP3 positive. Bone marrow dendritic cells were identified as CD11b CD11c positive and F4/80 negative. CD11b PerCP Cy5.5, CD11c E450, CD19 PE-Cy5, CD3 PE-Cy5, CD4 PE-Cy5, B220 PE, FoxP3 PE, F4/80 APC, and CD25 FITC were all purchased from eBioscience (San Diego, CA). 120g8 APC (BST2) was purchased from Imgenex (San Diego, CA). Surface expression of CD80 and CD86 on BMDCs was determined using flow cytometry. CD80 FITC and CD86 FITC were purchased from eBioscience. Flow cytometric analysis was performed using a FACScan flow cytometer (BD Biosciences, San Jose, CA), or a CYAN flow cytometer (Dako, Carpinteria, CA) and the data was analyzed using Summit (Dako, Carpinteria, CA, USA).

### Determination of HSA-specific immunoglobulins

HSA-specific immunoglobulins were determined as described previously [10]. Details are provided in the On Line Repository for this article.

### UV Inactivation of Bacteria

For UV-inactivation, chlamydial suspensions were placed under a UV lamp (15 W at 30 cm) for 15 min. UV-inactivation was confirmed by subculturing of treated bacteria in HEp-2 cells. *Bordetella bronchiseptica* (provided by Dr. Jeff Miller, UCLA, CA) was UV-inactivated as above.

### Bone marrow–derived DCs

Bone marrow–derived dendritic cells (BMDCs) were generated by incubating bone marrow cells with 10 ng/ml recombinant mouse GM-CSF (Biosource, Bethesda, MD) for 6 days, with medium changes at days 3 and 5. DCs were harvested at day 6 and purified with CD11c microbeads, as described above. Purity was assessed by means of flow cytometry was routinely around 98%.

### Cytokine detection

2×10^5^ mediastinal lymph node cells were stimulated with HSA (200 µg/ml) for three days and supernatants collected. IL-5 levels were determined by ELISA (eBioscience). BMDCs were incubated with either CP, LPS (InvivoGen, San Diego, CA), or LP2 (Pam2CSK4) (InvivoGen), and supernatants collected. Levels of IL-6 were determined by ELISA (BD Biosciences).

### Determination of HSA-specific immunoglobulins

For HSA-specific IgG1 and IgG2a titers, plates were coated with 50 µg/mL HSA overnight, followed by blocking with PBS/1% BSA at 37°C for 30 minutes. Plates were incubated with serum samples diluted in PBS/1% BSA at 37°C for 90 minutes, followed by detection of bound immunoglobulin with biotinylated anti-mouse IgG1 and IgG2a antibodies, respectively (BD Biosciences), and streptavidin (eBioscience). For HSA- specific IgE, HSA was biotinylated with the FluoreporterBiotin-XX kit (Invitrogen, Carlsbad, CA). Plates were coated with anti-mouse IgE antibody (BD Biosciences), blocked with BSA, and incubated with serum samples. Bound HSA-specific IgE was detected by using biotinylated HSA and streptavidin-HRP. As a standard, pooled sera from mice immunized with HSA plus LPS was used and set arbitrarily at 1.0 U/ml.

### Statistical analyses

Independent experiments were conducted at least in triplicate, except as otherwise noted. Data are reported as mean values ±SEM and compared by using 2-tailed unpaired Student's t test. For multiple comparison test, statistical significance was evaluated by one or two-way ANOVA with Tukey's post-hoc test where appropriate. A p value of less than 0.05 was required to reject the null hypothesis.

## Supporting Information

Figure S1 **Sensitization and challenge protocols.** **A:** CPUV protocol. Starting with day 0, groups of mice received intranasal injections of 100 µg HSA with or without 1×10^6^ UV-inactivated CP on 3 consecutive days (or LPS, or LP2). Control groups received HSA plus a mock extract of HEp-2 cells or CPUV only. At day 15, mice received 4 intranasal injections of 25 mg HSA. A control group received PBS. Mice were sacrificed 24 h after the final challenge. **B:** CP infection protocol. At day −5 either 5×10^5^ or 5×10^6^ IFU CP were injected intranasally to mice. 5 days later the mice were sensitized, challenged, and sacrificed as above. **C:** Depletion protocols. At day −5 either 5×10^5^ or 5×10^6^ IFU CP were injected intranasally to mice. Either diphtheria toxin, mAB 927, or mAB PC61 were injected i.p. into mice on the days indicated to deplete Tregs, plasmacytoid dendritic cells, and Tregs respectively. The mice were sensitized and challenged as before.
(TIF)

Figure S2 **UV-killed** ***Bordetella bronchiseptica*** **(BBUV) induces airway allergic sensitization to human serum albumin (HSA).** Mice were sensitized as indicated in Figure E1A. **A:** Inflammatory scores of H&E stained lung sections of mice after sensitization and challenge. **B:** BBUV-sensitized (n = 15) and PBS control (n = 9) eosinophil numbers per lung section area (mm^2^), representative peroxidase-stained sections (100-fold magnification) are shown. **C:** BBUV-sensitized (n = 9) and PBS control (n = 5) goblet cell numbers per basal membrane length (mm), representative periodic acid-Schiff-stained sections (100-fold magnification) are shown. **D:** HSA-specific IgE, IgG_1_, and IgG_2a_ relative titers. *p≤0.05, **p≤0.01, ***p≤0.001.
(TIF)

Figure S3 **Innate immune responses in TLR knockout BMDCs.** **A:** IL-6 expression in BMDCs from wild type, TLR2−/−, TLR4−/−, and MyD88−/− mice. DCs were infected with CP (MOI = 2.5), or exposed to CPUV (MOI2.5), LPS (100 ng/ml), or LP2 (100 ng/ml) for 24 hr. IL-6 was measured by ELISA in the supernatants. **B:** Up-regulation of costimulatory molecules on BMDCs from wild type, TLR2−/− and TLR4−/− mice. DCs were infected with CP (MOI = 2.5) and expression of CD80 and CD86 were determined by FACS after 24 h. Shown are data from uninfected controls (bold line), infected cells (dotted lines), and isotype controls (in grey).
(TIF)

Figure S4 **Quantization of Tregs and pDCs in the lung.** **A:** The total number of lung Tregs in wild type, TLR2−/−, and TLR4−/− uninfected and CP-infected mice. Wild type, TLR4−/−, and TLR2−/− mice (n = 3–6 per group) were inoculated with 5×10^5^ IFU of CP. 5 days after inoculation, mice were sacrificed, lung leukocyte preparations were generated and analyzed for the presence of CD4+, CD25+, Foxp3+ Tregs by flow cytometry. **B:** Tregs were assessed in the lungs of WT and TLR2−/− mice 10 days after low dose (5×10^5^) CP infection. Data are presented as the total count of CD4+, CD25+, FoxP3+ Tregs in the lung. **C–F:** WT mice were infected with either low dose (5×10^5^) or high dose (5×10^6^) CP intranasally. 5 days after infection, the lungs were harvested and single cell suspensions were analyzed by flow cytometry. **C:** Tregs. Data are presented as total count of CD4+, CD25+, FoxP3+ Tregs in the lung. **D:** pDCs. Data are presented as BST2+, B220+, CD3 CD19 CD11b- and side scatter low as a total pDC cell count in the lung. **E–F:** Representative Flow cytometric scatter plots for Tregs and pDCs in mouse Lungs during CP infection. **E:** Tregs. Data are presented as CD4+, CD25+, FoxP3+ Tregs. **F:** pDCs. Data are presented as BST2+, B220+, CD3 CD19 CD11b- and side scatter low. *p≤0.05, **p≤0.01, ***p≤0.001.
(TIF)
