# Supplementary figures and images for: Chlamydia pneumoniae Infection Induced Allergic Airway Sensitization Is Controlled by Regulatory T-Cells and Plasmacytoid Dendritic Cells
Source: PLoS One. 2011 Jun 10;6(6):e20784. doi: 10.1371/journal.pone.0020784 (PMC3112152; doi:10.1371/journal.pone.0020784)

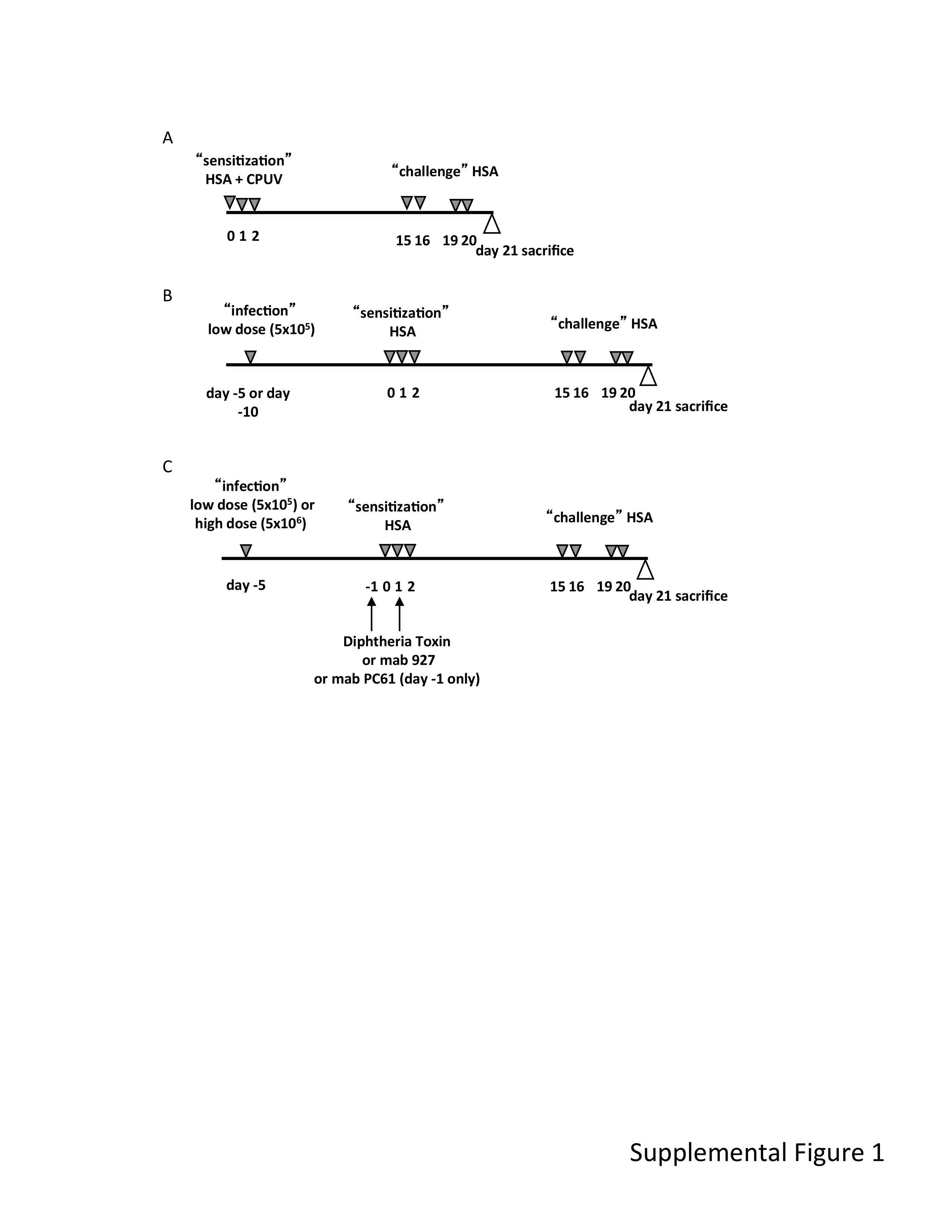

Supplement: Figure S1 — Sensitization and challenge protocols. A: CPUV protocol. Starting with day 0, groups of mice received intranasal injections of 100 µg HSA with or without 1×10^6^ UV-inactivated CP on 3 consecutive days (or LPS, or LP2). Control groups received HSA plus a mock extract of HEp-2 cells or CPUV only. At day 15, mice received 4 intranasal injections of 25 mg HSA. A control group received PBS. Mice were sacrificed 24 h after the final challenge. B: CP infection protocol. At day −5 either 5×10^5^ or 5×10^6^ IFU CP were injected intranasally to mice. 5 days later the mice were sensitized, challenged, and sacrificed as above. C: Depletion protocols. At day −5 either 5×10^5^ or 5×10^6^ IFU CP were injected intranasally to mice. Either diphtheria toxin, mAB 927, or mAB PC61 were injected i.p. into mice on the days indicated to deplete Tregs, plasmacytoid dendritic cells, and Tregs respectively. The mice were sensitized and challenged as before. (TIF) [file pone.0020784.s001.tif]

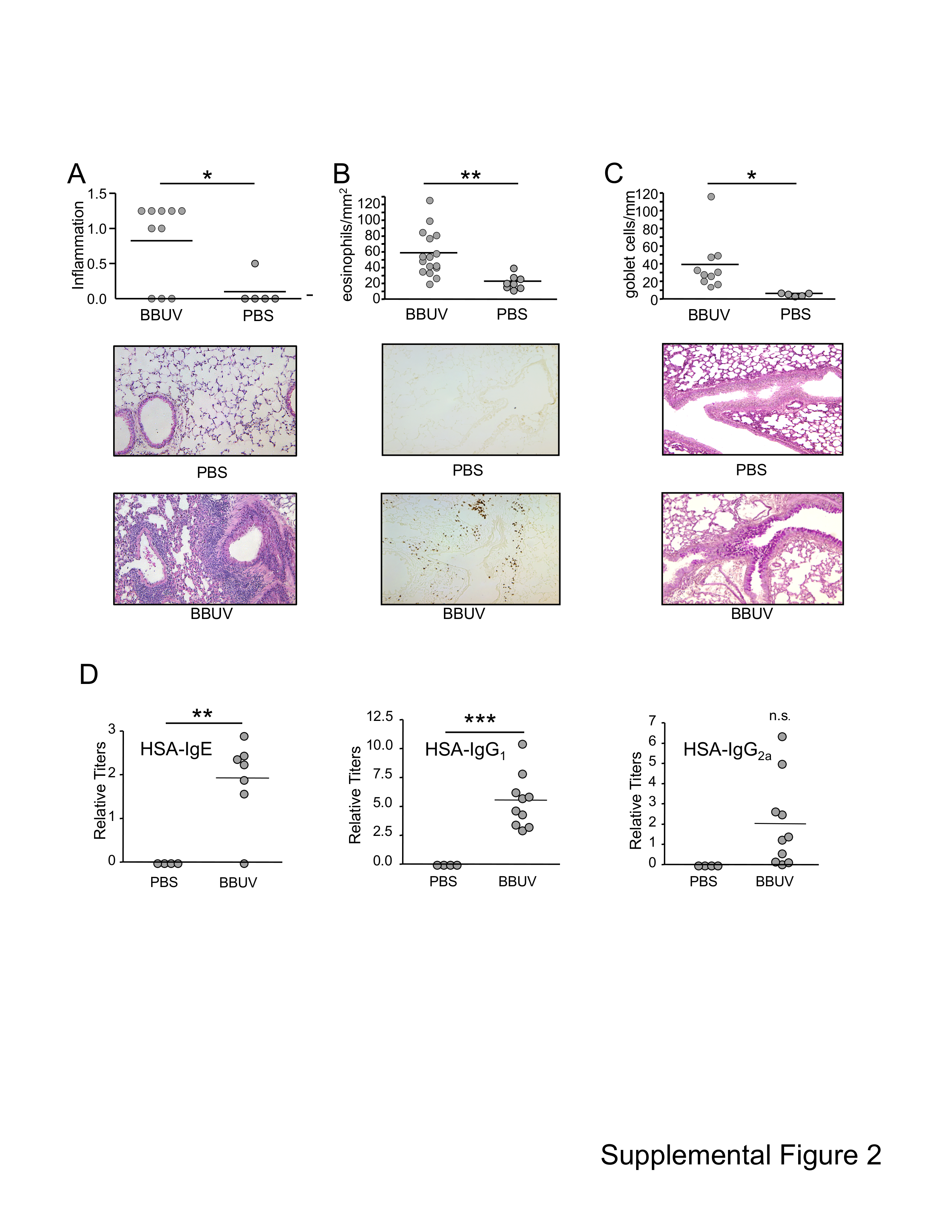

Supplement: Figure S2 — UV-killed Bordetella bronchiseptica (BBUV) induces airway allergic sensitization to human serum albumin (HSA). Mice were sensitized as indicated in Figure E1A. A: Inflammatory scores of H&E stained lung sections of mice after sensitization and challenge. B: BBUV-sensitized (n = 15) and PBS control (n = 9) eosinophil numbers per lung section area (mm2), representative peroxidase-stained sections (100-fold magnification) are shown. C: BBUV-sensitized (n = 9) and PBS control (n = 5) goblet cell numbers per basal membrane length (mm), representative periodic acid-Schiff-stained sections (100-fold magnification) are shown. D: HSA-specific IgE, IgG1, and IgG2a relative titers. *p≤0.05, **p≤0.01, ***p≤0.001. (TIF) [file pone.0020784.s002.tif]

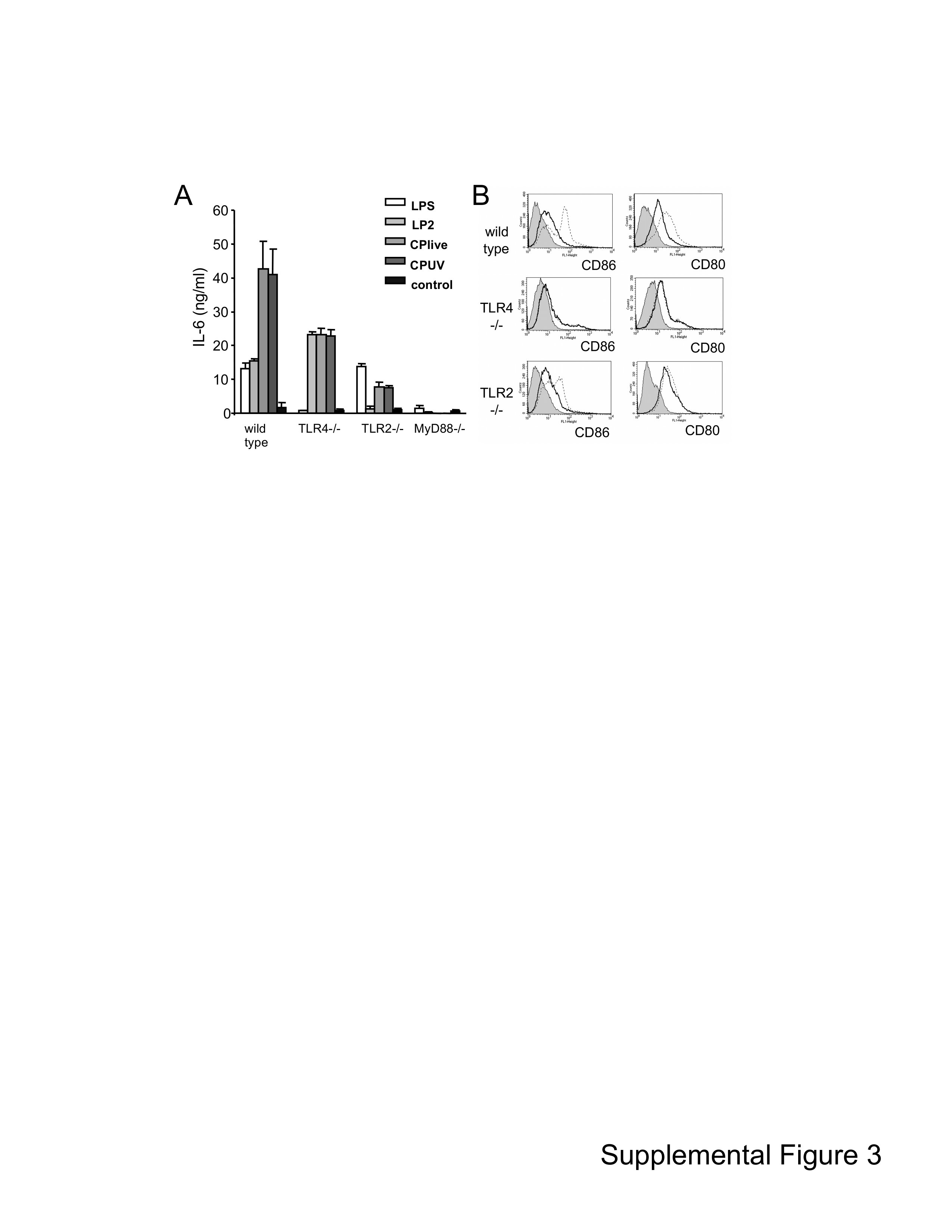

Supplement: Figure S3 — Innate immune responses in TLR knockout BMDCs. A: IL-6 expression in BMDCs from wild type, TLR2−/−, TLR4−/−, and MyD88−/− mice. DCs were infected with CP (MOI = 2.5), or exposed to CPUV (MOI2.5), LPS (100 ng/ml), or LP2 (100 ng/ml) for 24 hr. IL-6 was measured by ELISA in the supernatants. B: Up-regulation of costimulatory molecules on BMDCs from wild type, TLR2−/− and TLR4−/− mice. DCs were infected with CP (MOI = 2.5) and expression of CD80 and CD86 were determined by FACS after 24 h. Shown are data from uninfected controls (bold line), infected cells (dotted lines), and isotype controls (in grey). (TIF) [file pone.0020784.s003.tif]

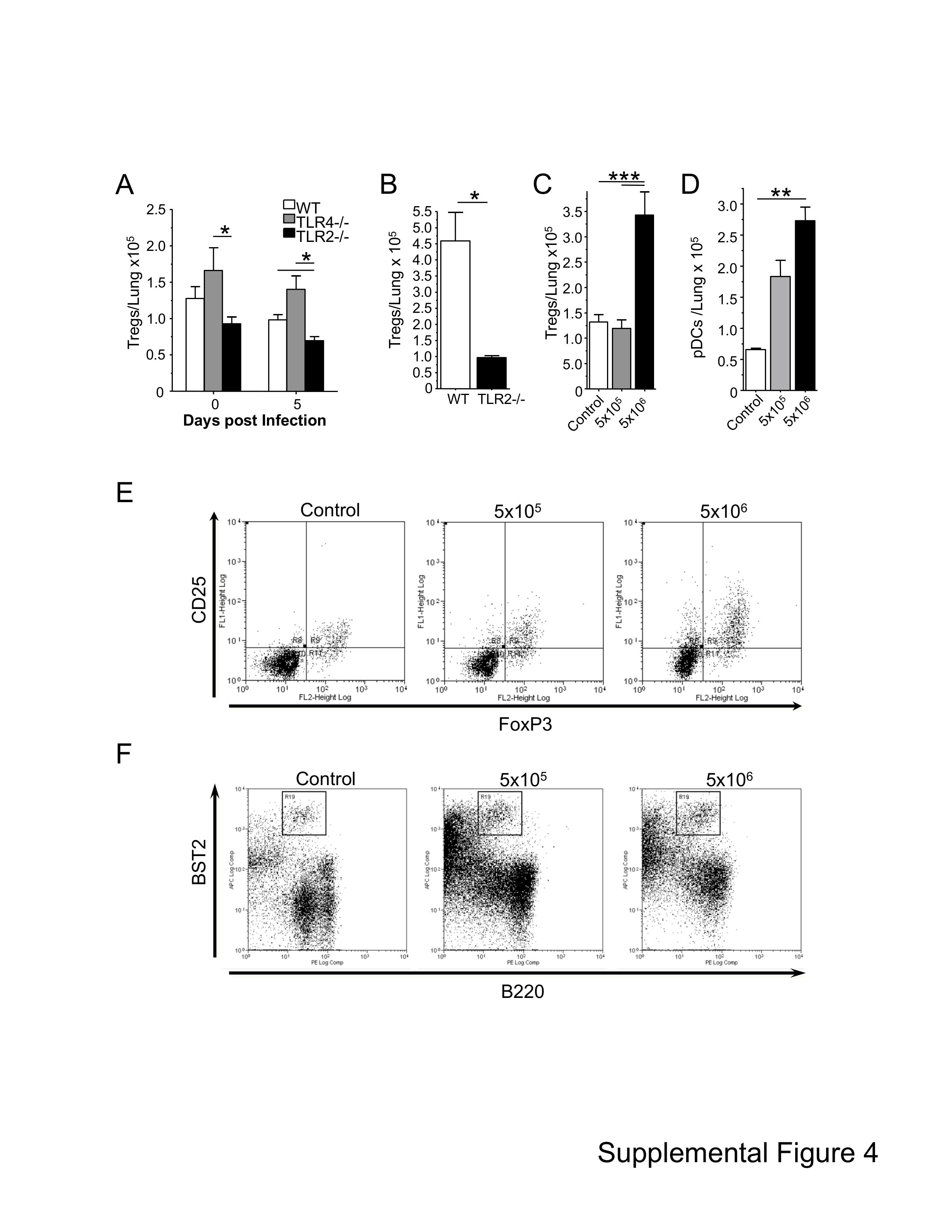

Supplement: Figure S4 — Quantization of Tregs and pDCs in the lung. A: The total number of lung Tregs in wild type, TLR2−/−, and TLR4−/− uninfected and CP-infected mice. Wild type, TLR4−/−, and TLR2−/− mice (n = 3–6 per group) were inoculated with 5×10^5^ IFU of CP. 5 days after inoculation, mice were sacrificed, lung leukocyte preparations were generated and analyzed for the presence of CD4+, CD25+, Foxp3+ Tregs by flow cytometry. B: Tregs were assessed in the lungs of WT and TLR2−/− mice 10 days after low dose (5×10^5^) CP infection. Data are presented as the total count of CD4+, CD25+, FoxP3+ Tregs in the lung. C–F: WT mice were infected with either low dose (5×10^5^) or high dose (5×10^6^) CP intranasally. 5 days after infection, the lungs were harvested and single cell suspensions were analyzed by flow cytometry. C: Tregs. Data are presented as total count of CD4+, CD25+, FoxP3+ Tregs in the lung. D: pDCs. Data are presented as BST2+, B220+, CD3 CD19 CD11b- and side scatter low as a total pDC cell count in the lung. E–F: Representative Flow cytometric scatter plots for Tregs and pDCs in mouse Lungs during CP infection. E: Tregs. Data are presented as CD4+, CD25+, FoxP3+ Tregs. F: pDCs. Data are presented as BST2+, B220+, CD3 CD19 CD11b- and side scatter low. *p≤0.05, **p≤0.01, ***p≤0.001. (TIF) [file pone.0020784.s004.tif]
